# Realist review protocol on user involvement in complex interventions in health and social care research among populations experiencing exclusion

**DOI:** 10.1136/bmjopen-2025-114512

**Published:** 2026-08-11

**Authors:** Sophie Nadia Gaber, Penny Rapaport, Luke Johnson, Jenny Eriksson Lundström, Åsa Kneck, Elisabet Mattsson, Rikke Siersbaek

**Affiliations:** 1 CIRCLE – Complex Intervention Research in Health and Care, Department of Women's and Children’s Health, Uppsala University, Uppsala, Sweden; 2 Division of Psychiatry, University College London, London, UK; 3 Collaborative Centre for Inclusion Health, Department of Epidemiology and Public Health, University College London, London, UK; 4 Center for Inclusion Health, Allegheny Health Network, Pittsburgh, Pennsylvania, USA; 5 Department of Health Care Sciences, Marie Cederschiöld University College, Stockholm, Sweden; 6 School of Medicine, Trinity College Dublin, Dublin, Ireland

**Keywords:** Psychosocial Intervention, Health Equity, Patient Participation, Delivery of Health Care, Integrated

## Abstract

**Abstract:**

**Introduction:**

User involvement is important for improving accessibility, quality and equity in health and social care research. However, the specific mechanisms by which it contributes to, or detracts from intervention outcomes are unclear, particularly for populations experiencing exclusion and health and social inequities in high-income countries. This realist review aims to understand and describe key mechanisms of user involvement in complex interventions in health and social care research among populations experiencing exclusion in high-income countries. Specifically, we will explore how, why, for whom and under what circumstances user involvement contributes to complex interventions in health and social care research among populations experiencing exclusion.

**Methods and analysis:**

We will conduct a six-step realist review: (1) clarifying the scope, (2) searching for evidence, (3) selecting and appraising sources, (4) extracting and organising data, (5) synthesising data and (6) refining the synthesis. We will systematically review and synthesise relevant sources from academic databases and grey literature. Workshops will be held with experts who have lived experience of exclusion and/or professionals working in inclusion health to gather feedback and refine the findings. A group of external academic experts in the field will also be consulted. Following Pawson and Tilley’s iterative approach, data will be extracted, analysed and synthesised into explanatory theoretical findings and reported in accordance with the Realist And Meta-narrative Evidence Syntheses Evolving Standards.

**Ethics and dissemination:**

Formal ethical approval is not required for this study as it is a review of published literature. Our overall dissemination strategy will be developed in collaboration with the experts who have lived experience of exclusion, as well as three professionals working in inclusion health. Findings will be shared with these expert contributors and disseminated through various channels, including a peer-reviewed publication, lay summary report and conference presentations.

**PROSPERO registration number:**

CRD420251046835.

**STRENGTHS AND LIMITATIONS OF THIS STUDY**

- Incorporates ongoing input from experts with lived experience of exclusion and/or professionals working in inclusion health, as well as external academic experts.
- Integrates a variety of evidence sources and study designs from peer-reviewed literature, grey literature and funded research databases to develop and refine context–mechanism–outcome configurations.
- Includes independent screening, data extraction and quality appraisal by at least two reviewers.
- Uses interpretive judgement to develop context–mechanism–outcome configurations, which may introduce subjectivity.
- Restricts the review to English-language and Swedish-language publications from high-income countries, which may limit the transferability of the findings.

## Introduction

 Rising health and social inequities underscore the need for inclusion health—a service, research and policy agenda that seeks to prevent and redress these inequities among those experiencing exclusion.^1 2^ This review focuses on populations experiencing exclusion who are typically the focus of inclusion health. Exclusion can be conceptualised in multiple ways and operates across several social, structural and health-related dimensions. Based on inclusion health policy^3^ and literature,^2 4 5^ we include the following groups which often overlap intersectionally: people experiencing homelessness, people who are currently incarcerated and/or have a history of contact with the criminal justice system, people who use drugs (including those with a substance use disorder), people with hazardous or harmful drinking (including those with an alcohol use disorder),^6^ sex workers, people held in immigration detention settings, vulnerable and/or forced migrants (asylum seekers, people who have been refused asylum, refugees and undocumented migrants), victims of modern slavery and human trafficking, and Romany Gypsy, Roma and Traveller communities. These populations experience social and material deprivation with complex, overlapping health and social care needs.^1 4 7^ Compared with the general population, in high-income countries, people experiencing exclusion have a 12-fold and 8-fold increase in all-cause mortality for women and men, respectively.^2^

For populations experiencing exclusion, barriers to care are prevalent, including stigma, insufficient person-centred approaches, fragmented and under-resourced services and a lack of adequate professional support.^8–10^ The constant need to secure essentials like food or shelter often takes priority and hinders access to health and social care needs, while previous negative experiences with services may deter individuals from seeking care.^8 9^ Populations experiencing exclusion account for considerable and far-reaching individual and societal costs associated with substance/drug use, the criminal justice system, unemployment, housing assistance, social benefits, premature deaths and chronic health conditions.^4 11^ Despite disproportionately poor health outcomes, populations experiencing exclusion are frequently overlooked in public health research, policy and investment.^4^ In research, this has been referred to as ‘intervention-generated inequality’, where interventions fail to capture the needs and preferences of such populations leading to and further deepening health and social inequities.^12^ This exclusion exacerbates sample bias,^12^ limits generalisability^13^ and undermines ethical fundamentals.^14 15^ Beyond ethical imperatives, such exclusion undermines commitment to high-quality, inclusive health and social care services.^16 17^

Over recent decades, health research has increasingly moved from viewing patients and communities as passive research participants towards recognising them as active contributors to the production of knowledge. This shift has been driven by patient rights movements, participatory research traditions and growing recognition that research is more relevant, ethical and applicable when informed by the experiences and priorities of those most affected.^18^ Inclusive approaches have also been supported by complexity-informed perspectives, which highlight that health interventions are embedded within dynamic social systems and that meaningful understanding of these systems requires engagement with the perspectives, relationships and experiences of diverse interest-holders.^19^ Rather than treating knowledge as solely generated by researchers, participatory and community-based approaches emphasise the co-construction of knowledge and seek to address power imbalances that influence whose voices are heard and valued.^19^ More recently, concerns regarding epistemic justice have further highlighted the importance of recognising people experiencing social exclusion not only as research participants but as legitimate knowers whose experiential knowledge can contribute to understanding and improving health and social care systems.^20 21^ Despite this growing emphasis on inclusive research, meaningful involvement of populations experiencing exclusion remains challenging, and evidence regarding how involvement contributes to intervention development, implementation and outcomes remains limited.

Alongside growing recognition of the importance of inclusive and participatory approaches to research, substantial health and social inequities persist among populations experiencing exclusion. In high-income countries, there are increasing calls for more integrated health and social care services that address their complex and interconnected needs.^2 4^ Oversimplified approaches to inclusion health run the risk of overlooking comorbidity and social determinants of health, leading to missed opportunities for prevention, diagnosis and management.^2 4^ Populations experiencing exclusion may require a more holistic and multicomponent approach to managing their health than a simple intervention addressing one issue is able to provide (eg, treating hepatitis C, substance use, hypertension, malnutrition etc all together and seeing it as one package).

Complex interventions have traditionally been defined as interventions comprising multiple interacting components.^22^ More recent conceptualisations recognise that complexity arises not only from the intervention itself but also from the dynamic social systems in which it is implemented, the context-dependent nature of outcomes and the interactions between individuals, organisations and wider systems.^23–25^ Health and social care interventions are inherently complex, involving many interacting people, structures and components.^25^ Realist approaches are therefore particularly well suited to understanding complex interventions because they explain how and why outcomes arise through interactions between contexts and underlying causal mechanisms rather than isolating intervention effects from the settings in which they occur.^19^

Community engagement is increasingly emphasised as an important component of ethical health research, contributing to respectful engagement with individuals and communities, building trust and social relationships, acknowledging vulnerabilities and researcher obligations, supporting informed consent and promoting other ethically appropriate research processes.^26^ Much of this literature has emerged from global health research; however, these principles are also relevant to research involving populations experiencing exclusion in high-income countries. Internationally, the importance of meaningful user involvement of patients and the public in health and social care research is emphasised.^17^ While there is a lack of consensus in terminology, guidelines emphasise research ‘with’ or ‘by’ the public, rather than ‘to’, ‘about’ or ‘for’ them, highlighting the potential benefits of user involvement in research.^27 28^ User involvement has the potential to enhance intervention acceptability, accessibility and relevance, which may promote person-centred approaches and improved health outcomes.^12^ It may also contribute to more inclusive and equitable forms of knowledge production by incorporating the experiential expertise of people directly affected by health and social care interventions.^18 20^

Compared with an earlier broader rapid realist review,^29^ this will be the first review, to our knowledge, to focus on user involvement in complex interventions in health and social care research among populations experiencing exclusion. This review’s originality lies in synthesising key underlying causal mechanisms driving user involvement with populations experiencing exclusion, offering insights into how and why such involvement works or does not work for these populations in real-world contexts. In summary, user involvement is widely acknowledged, but the specific dynamics of how it operates for populations experiencing exclusion are underexplored.

## Aim and research questions

This realist review aims to understand and describe key mechanisms of user involvement in complex interventions in health and social care research among populations experiencing exclusion in high-income countries. Specifically, we will explore how, why, for whom and under what circumstances user involvement contributes to complex interventions in health and social care research among populations experiencing exclusion. To achieve this aim, we seek to address the following research questions:

### Primary research question

How does user involvement contribute to complex interventions in health and social care research among populations experiencing exclusion? In what ways, for what reasons and for whom?

### Secondary research questions

1. What types of user involvement have been used in complex interventions in health and social care research among populations experiencing exclusion?
2. Which contextual conditions facilitate user involvement with populations experiencing exclusion to achieve the intended outcomes?

## Methods and analysis

### Study design

The study will run from 1 July 2025, when funding was first received to establish the project and review team, through 30 June 2026, with additional time allocated for dissemination. We will perform a realist review, an established systematic literature review approach. Online supplemental file 1 provides a glossary of key terms used in the realist review. Realist reviews follow systematic procedures to explore the mechanisms underpinning complex interventions in health and social care research in varied contexts.^30 31^ They are well-suited to address the heterogeneity of user involvement in complex health and social interventions by synthesising diverse sources.^31 32^

We aim to include approximately 8–10 experts who have lived experience of exclusion and/or professionals working in inclusion health who will be involved throughout the research process. The final number will depend on availability and willingness to participate. Their contribution will include expert consultation and workshops, as well as informal consultations when needed. However, they will not be involved as research participants. Potential members will be approached through the authors’ ongoing collaborations and extended networks across inclusion health services and organisations. They will attend voluntarily and be compensated as per best practice guidance.^33^ We will foster trust and avoid perpetuating power imbalances, using person-first and non-technical language. There is a low likelihood, but if needed, psychosocial support will be provided in case individuals feel provoked by any discussions.

Realist ontology is based on the view that underlying causal mechanisms (M) are triggered in specific contexts (C) to produce certain outcomes (O). In realist reviews, these are expressed as Context-Mechanism-Outcome-configurations (CMOCs) and they are the building blocks of programme theories.^30^ A programme theory can be defined as an explanation or hypothesis of how and why a programme (ie, intervention) is expected to work, given contextual influences and underlying mechanisms of action (see online supplemental file 1).^31^ Our programme theories will address our research questions, providing a deeper understanding of key underlying causal mechanisms of user involvement with populations experiencing exclusion in complex interventions in health and social care research. The realist review approach is suited to our research questions as it is highly complex in nature and incorporates user perspectives in the process.

**Figure 1:**
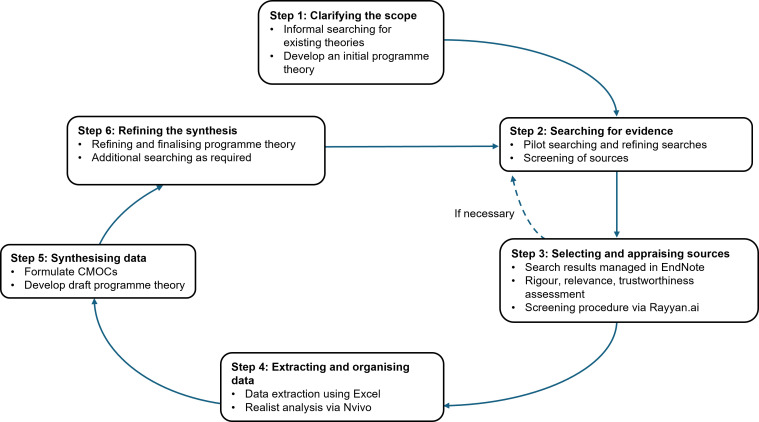
Steps of the realist review.

Using procedures described in earlier literature,^34 35^ we will perform six iterative steps. Figure 1 shows the review process: (1) clarifying the scope, (2) searching for evidence, (3) selecting and appraising sources, (4) extracting and organising data, (5) synthesising data and (6) refining the synthesis. The review process and findings will be reported according to the Realist And Meta-narrative Evidence Syntheses: Evolving Standards (RAMESES) publishing standards (online supplemental file 2).^36^

#### Step 1: clarifying the scope

In preparation for the literature search, an initial programme theory will be developed based on informal reading of literature and discussions between the review team (ie, authors) and experts who have lived experience of exclusion and/or professionals working in inclusion health. The initial programme theory will be informed by the review team’s initial understanding of the subject and will guide the selection of search terms. Workshops with experts who have lived experience of exclusion and/or professionals working in inclusion health will be held to support the development of the initial programme theory.

#### Step 2: searching for evidence

Database searches: We will perform systematic searches of academic databases (MEDLINE, PsycINFO, CINAHL, Web of Science, Cochrane Library). The search strategy will be developed in consultation with a librarian at Uppsala University Library and reviewed by another librarian. The strategy will be designed for MEDLINE using MeSH terms and then adapted to the other databases. The search will include relevant Subject Headings when possible, and terms are searched for in the title/abstract of publications. Search strings will be iteratively developed and piloted, using indexing terms and keywords related to populations experiencing exclusion, user involvement and complex interventions. Search terms will be determined based on the initial programme theory and the a priori protocol, with input from the scientific librarians. We will also search reference lists and citations of included articles and review those published by the lead authors of included articles. A comprehensive list of search terms and databases will be included in subsequent publications on completion of the planned review.

Grey literature: We will search relevant grey literature sources (eg, Google Scholar and websites of organisations that work with populations experiencing exclusion). Funded research projects: We will screen Swedish-funded projects (via the Swecris database) and Swedish-based research publications (via the SwePub database), promoting comprehensive coverage of relevant research outputs.

Throughout the review, academic and grey literature searches will be updated as needed to address any research gaps. The group of external academic experts will be consulted to identify any additional sources that meet inclusion criteria. Potential members of this group of external academic experts will be approached by the authors of this review based on their respective networks. Consultations with the external academic experts will be conducted through online meetings with the review team and via ongoing communication and updates.

#### Step 3: selecting and appraising sources

Search results will be managed in EndNote (V.21). After de-duplication, remaining sources will be screened by lead author (SNG) at the title and abstract and full-text stages. At both stages, a random sample of 10% of articles will be independently screened by a second author (EM or RS) to ensure accuracy. Discrepancies will be resolved through discussions with a third author (EM or RS). Those that meet the eligibility criteria presented in table 1 will undergo full text screening. Homelessness was defined according to the categories outlined in the European Typology of Homelessness and Housing Exclusion.^37^

**Table 1:** Inclusion and exclusion criteria

	Inclusion	Exclusion
Population	Populations experiencing exclusion (≥18 years), as described in previous literature^2–4^	<18 years Determined as a population outside of our definition of populations experiencing exclusion in relation to the inclusion health service, research and policy agenda
Intervention	Any type of complex interventions in health and social care research that: Included user involvement in any phase described by the Medical Research Council framework for complex interventions^24^: developing and/or identifying complex interventions, testing feasibility, evaluation, implementation	Complex interventions targeting populations experiencing exclusion but without user involvement
Context	High-income countries, as defined by the World Bank^41^	Low- and middle-income countries
Type of publication	Published in English or Swedish since 2005–present Any study design or publication type, including grey literature and other sources (eg, Swecris and SwePub)	Studies published in other languages or before 2005

Screening will be performed in Rayyan.ai. Formal quality assessment checklists are not typically used in realist reviews, as the focus is on whether evidence is sufficiently robust and meaningful to inform theory development rather than on standardised appraisal criteria. Consistent with previous realist reviews,^38^ sources will be appraised for rigour (use of appropriate methods and plausibility of findings), relevance (contribution to the research questions) and trustworthiness (credibility of data). Only studies relevant to the review question and contributing insights to its aim will be included.

#### Step 4: extracting and organising data

Data from the included sources will be collated onto a data extraction form in NVivo (V.12) or another format (eg, Microsoft Word, Excel) based on RAMESES publication standards.^36^ The lead author (SNG) will extract and code the data using a theory-driven realist approach, combining inductive, deductive and retroductive coding to identify patterns of causality. From there, CMOCs will be developed, grounded in the data. These CMOCs will then be refined and synthesised into an overarching programme theory that captures broader causal patterns and can be applied across similar contexts.

Using realist approaches to analysis in the school of Pawson and Tilley,^30 39^ the lead author will inductively code data from sources assessed as most useful in terms of rigour, relevance and trustworthiness in the earlier screening stages (ie, sources with rich data that adhered to the chosen methodology) as these are likely to contribute most to the development of the CMOC and programme theory. Next, these codes will be applied deductively to additional sources with new codes added as needed. Codes will be assigned under conceptual labels that capture what the concept is about.^40^

Together with the review team, the lead author will analyse the data retroductively. Retroductive analysis is a distinctive feature of realist analysis used to identify and explain the underlying causal patterns within the data. This process involves exploring relationships between different aspects of the data and configuring these into CMOCs. Each CMOC represents the reviewers’ reasoned inference about the hidden causal mechanisms that generate particular outcomes within specific contexts.^40^

#### Step 5: synthesising data

Once initial codes are developed, draft CMOCs will be generated by the lead author (SNG). These will be discussed within the review team and in consultation with external academic experts. Each CMOC will remain closely tied to the data, and multiple sources will inform each configuration. CMOCs will be iteratively refined and combined to develop a programme theory that seeks to explain key mechanisms of user involvement in complex interventions in health and social care research among populations experiencing exclusion.

#### Step 6: refining the synthesis

The review team will scrutinise and iteratively refine the CMOCs, discussing interpretations and relevance and drafting summaries to show how each CMOC was derived from coded data (eg, a Word document listing CMOCs with supporting data and excerpts). The draft programme theory, CMOCs, and summaries will be presented and discussed with experts who have lived experience of exclusion and/or professionals working in inclusion health, potentially through workshops or other preferred formats. The intention is to collaboratively refine and enhance the relevance of the draft programme theory, CMOCs and summaries, as well as to develop potential recommendations. Based on this collaborative process, the programme theory will be further refined through iterative re-reading of sources and ongoing team discussions, as necessary. Additional literature searches will be conducted by one of the reviewers (ie, the lead author) if required.

## Ethics and dissemination

No formal ethics approval will be required or sought, as the realist review will use only previously published data. The dissemination strategy will be developed collaboratively with experts who have lived experience of exclusion and/or professionals working in inclusion health, as well as external academic experts, to promote relevance and accessibility to diverse audiences. The findings will be shared with these expert contributors. Initial proposals include dissemination to both academic and non-academic audiences for broad impact. The findings will be shared through an open-access, peer-reviewed scientific article featuring key recommendations and presented at national and/or international conferences targeting audiences in health and social care research, policy and practice. Additionally, an online seminar will be hosted to share the realist review process and findings with academic staff and students in related fields. Moreover, a summary report will be developed based on the review findings and discussions with experts who have lived experience of exclusion and/or professionals working in inclusion health. The review team will draw on their experience in popular science communication to enhance reach and engagement.

## Supplementary material

10.1136/bmjopen-2025-114512 online supplemental file 1

10.1136/bmjopen-2025-114512 online supplemental file 2
