# Supplementary material for: Realist review protocol on user involvement in complex interventions in health and social care research among populations experiencing exclusion
Source: BMJ Open. 2026 Aug 11;16(8):e114512. doi: 10.1136/bmjopen-2025-114512 (PMC13475212; doi:10.1136/bmjopen-2025-114512)
Supplement: Supplementary file 1 [file bmjopen-16-8-s002.pdf]

Supplementary file 2. RAMESES publication standards: realist syntheses

**TITLE**			**Reported in document Y/N/Unclear**	**Page(s) in document**
1		In the title, identify the document as a realist synthesis or review	Y	1
ABSTRACT				
2		While acknowledging publication requirements and house style, abstracts should ideally contain brief details of: the study's background, review question or objectives; search strategy; methods of selection, appraisal, analysis and synthesis of sources; main results; and implications for practice.	Y (but main results and implications for practice not reported for protocol)	1
INTRODUCTION				
3	Rationale for review	Explain why the review is needed and what it is likely to contribute to existing understanding of the topic area.	Y	2-3
4	Objectives and focus of review	State the objective(s) of the review and/or the review question(s). Define and provide a rationale for the focus of the review.	Y	3
METHODS				

TITLE			Reported in document Y/N/Unclear	Page(s) in document
5	Changes in the review process	Any changes made to the review process that was initially planned should be briefly described and justified.	N/A	N/A
6	Rationale for using realist synthesis	Explain why realist synthesis was considered the most appropriate method to use.	Y	3
7	Scoping the literature	Describe and justify the initial process of exploratory scoping of the literature.	Y (includes plan for scoping the literature only as it is a protocol)	3-4
8	Searching processes	While considering specific requirements of the journal or other publication outlet, state and provide a rationale for how the iterative searching was done. Provide details on all the sources accessed for information in the review. Where searching in electronic databases has taken place, the details should include, for example, name of database, search terms, dates of coverage and date last searched. If individuals	Y (includes plan for searching processes only as it is a protocol)	4

TITLE			Reported in document Y/N/Unclear	Page(s) in document
		familiar with the relevant literature and/or topic area were contacted, indicate how they were identified and selected.		
9	Selection and appraisal of documents	Explain how judgements were made about including and excluding data from documents, and justify these.	Y (includes plan for selection and appraisal of documents only as it is a protocol)	4-5
10	Data extraction	Describe and explain which data or information were extracted from the included documents and justify this selection.	Y (includes plan for data extraction only as it is a protocol)	5
11	Analysis and synthesis processes	Describe the analysis and synthesis processes in detail. This section should include information on the constructs analyzed and describe the analytic process.	Y (includes plan for analysis and synthesis processes only as it is a protocol)	5
RESULTS				
12	Document flow diagram	Provide details on the number of documents assessed for eligibility and included in the review with reasons for exclusion at each stage as well as an indication of their source of	N/A (PRISMA diagram will be included in final realist review)	N/A

TITLE			Reported in document Y/N/Unclear	Page(s) in document
		origin (for example, from searching databases, reference lists and so on). You may consider using the example templates (which are likely to need modification to suit the data) that are provided.		
13	Document characteristics	Provide information on the characteristics of the documents included in the review.	N/A	N/A
14	Main findings	Present the key findings with a specific focus on theory building and testing.	N/A	N/A
DISCUSSION				
15	Summary of findings	Summarize the main findings, taking into account the review's objective(s), research question(s), focus and intended audience(s).	N/A	N/A
16	Strengths, limitations and future research directions	Discuss both the strengths of the review and its limitations. These should include (but need not be restricted to) (a) consideration of all the steps in the review process	Y (full presentation of strengths, limitations and future directions will be included in the final realist review)	1

TITLE			**Reported in document Y/N/Unclear**	**Page(s) in document**
		and (b) comment on the overall strength of evidence supporting the explanatory insights which emerged.<br><br>The limitations identified may point to areas where further work is needed.		
17	Comparison with existing literature	Where applicable, compare and contrast the review's findings with the existing literature (for example, other reviews) on the same topic.	N/A	N/A
18	Conclusion and recommendations	List the main implications of the findings and place these in the context of other relevant literature. If appropriate, offer recommendations for policy and practice.	N/A	N/A
19	Funding	Provide details of funding source (if any) for the review, the role played by the funder (if any) and any conflicts of interests of the reviewers.	Y	6
