# Supplementary material for: Realist review protocol on user involvement in complex interventions in health and social care research among populations experiencing exclusion
Source: BMJ Open. 2026 Aug 11;16(8):e114512. doi: 10.1136/bmjopen-2025-114512 (PMC13475212; doi:10.1136/bmjopen-2025-114512)
Supplement: Supplementary file 2 [file bmjopen-2025-114512.reviewer_comments.pdf]

PEER REVIEW HISTORY

BMJ Open publishes all reviews undertaken for accepted manuscripts. Reviewers are asked to complete a checklist review form and are provided with free text boxes to elaborate on their assessment. These free text comments are reproduced below.

ARTICLE DETAILS

Title (Provisional)

A realist review protocol on user involvement in complex interventions in health and social care research among populations experiencing exclusion

Authors

Gaber, Sophie Nadia; Rapaport, Penny; Johnson, Luke; Eriksson Lundström, Jenny; Kneck, Åsa; Mattsson, Elisabet; Siersbaek, Rikke

---

VERSION 1 - REVIEW

**Reviewer**	**1**
**Name**	**Adhikari, Bipin**
**Affiliation**	**Mahidol Oxford Tropical Medicine Research Unit**
**Date**	**11-Jun-2026**
**COI**	**None**

This is an interesting protocol emphasizing user involvement in complex interventions in health and social care research among populations experiencing exclusion.

Broad comment:

Relate and discuss with how community engagement as well addresses the inclusion/involvement for ethical global health research. This way, you broaden your relevance and strengthen your case/rationale.

More: Adhikari B, Pell C, Cheah PY. Community engagement and ethical global health research. Glob Bioeth. 2019 Dec 20;31(1):1-12. doi: 10.1080/11287462.2019.1703504. PMID: 32002019; PMCID: PMC6968663.

Discuss more about the inclusivity in health research, historical development (I think there is an opportunity for this) and why has it been increasingly emphasized, again to enhance the broad readership.

Explain what is complex interventions in health and social care research. Realist methods is well-suited for such complex interventions, but you have not discussed about what complex interventions are.

More: Byrne D, Callaghan G: Complexity theory and the social sciences: The state of the art. Routledge; 2022.

Minor:

Line 162: how many experts are you aiming for? Give a tentative sample here.

Line 292-296: Is the dissemination planned for the participants as well?

VERSION 1 - AUTHOR RESPONSE

COMMENTS TO AUTHORS	RESPONSE
**Reviewer 1 comments:**	
- This is an interesting protocol emphasizing user involvement in complex interventions in health and social care research among populations experiencing exclusion.	Thank you for your thorough review of the manuscript. We appreciate your recognition that this is an interesting protocol.
**Broad comment:**	
- Relate and discuss with how community engagement as well addresses the inclusion/involvement for ethical global health research. This way, you broaden your relevance and strengthen your case/rationale. More: Adhikari B, Pell C, Cheah PY. Community engagement and ethical global health research. Glob Bioeth. 2019 Dec 20;31(1):1-12. doi: 10.1080/11287462.2019.1703504. PMID: 32002019; PMCID: PMC6968663.	We appreciate the reviewer highlighting the broader literature on community engagement in ethical health research. While the focus of our realist review is on user involvement in health and social care research in high-income countries, we agree that this literature provides important conceptual context. We have therefore expanded the Introduction to acknowledge the contribution of community engagement to ethical research and have cited the suggested paper. **[See Introduction, p. 8 lines 170-177]**
- Discuss more about the inclusivity in health research, historical development (I think there is an opportunity for this) and why has it been increasingly emphasized, again to enhance the broad readership.	We agree that additional context regarding the development of inclusive health research would benefit readers who are less familiar with this field. We have therefore expanded the introduction to briefly describe the historical shift from researcher-led approaches towards participatory and inclusive models of health research, including the growing recognition of patients' and communities' rights to influence research that affects them. We also highlight how concerns regarding equity, representativeness, research relevance, and the ethical imperative to reduce the exclusion of marginalised populations have contributed to increasing emphasis on inclusive research practices internationally. To maintain the focus and conciseness of the introduction, we have added a brief contextual paragraph and relevant references rather than a detailed historical overview. **[See Introduction, p. 6 lines 122-139 & p. 7 lines 140-148, p. 8 line 183-186]**
- Explain what is complex interventions in health and social care research. Realist methods is well-suited for such complex	We agree that the manuscript would benefit from a clearer definition of complex interventions. We have revised the Introduction to include a definition of complex interventions and to explain

interventions, but you have not discussed about what complex interventions are.<br>More: Byrne D, Callaghan G: Complexity theory and the social sciences: The state of the art. Routledge; 2022.	how a complexity-informed perspective emphasises the dynamic interactions between intervention components, contexts and human agency. We have also incorporated the suggested reference by Byrne and Callaghan to strengthen the rationale for using realist methods to evaluate complex interventions.<br>**[See Introduction, p. 7 lines 155-162 & p. 8 lines 163-164]**
**Minor:**	
- Line 162: how many experts are you aiming for? Give a tentative sample here.	Thank you for this helpful suggestion. We have clarified that we aim to include 8–10 experts with lived experience of exclusion, while recognising that the final number will depend on availability and willingness to participate.<br>**[See Study design, p. 10 lines 223-226]**
- Line 292-296: Is the dissemination planned for the participants as well?	We appreciate the reviewer highlighting the importance of sharing the findings with those who contribute to the review. We have revised the text to clarify that the findings will be shared with the experts with lived experience of exclusion and/or professionals working in inclusion health, as well as external academic experts, who contribute to the dissemination strategy.<br>**[See Ethics and dissemination in the Abstract p. 3 lines 47-48 & p. 16 lines 356-358]**
