# Supplementary material for: Realist review protocol on user involvement in complex interventions in health and social care research among populations experiencing exclusion
Source: BMJ Open. 2026 Aug 11;16(8):e114512. doi: 10.1136/bmjopen-2025-114512 (PMC13475212; doi:10.1136/bmjopen-2025-114512)
Supplement: Supplementary file 3 [file bmjopen-16-8-s001.docx]

**Supplementary file 1.** Glossary of key terms used in the realist review

| **Term** | **Explanation** |
| --- | --- |
| User involvement | Internationally, terminology for user involvement is not always used consistently in research, and other commonly used terms include *patient and public involvement*, *engagement,* and *participation*. This review focuses on *involvement* as defined by the National Institute for Health and Care Research (NIHR).^1^ The NIHR describes *involvement* as research carried out with or by patients and the public, rather than to, about or for them.  User involvement emphasises working collaboratively with service users, patients, and the public, and sharing decision-making. This differs from:  (i) engagement, which focuses on raising awareness and sharing research knowledge and findings, and  (ii) participation, which involves people giving formal consent and taking part in a research study or trial. |
| Context | Elements in the surrounding environment of a programme (i.e., intervention) that influence its outcomes (e.g., demographics, legislation, cultural norms).^2, 3^ |
| Mechanism | Resources a programme (i.e., intervention) provides and the ways in which people respond to these resources (e.g., information, advice, trust, engagement, motivation).^2^ |
| Outcome | Intended or unintended effects that result from context–mechanism interactions (e.g., changes in behaviour, service uptake, decision-making, resilience, health outcomes, self-efficacy, social connections).^2^ |
| Context-Mechanism-Outcome configuration (CMOC) | The CMOC is used in realist analysis to identify how different elements of the context can either trigger or hinder mechanisms (i.e., keep latent).^2^ The CMOC provides a basic framework for realist causal explanation and can guide the review process, from theory development to data collection protocols and data analysis. These theories are then tested against the available evidence and confirmed, challenged, or refined.^2, 4^ |
| Programme theory | A realist programme theory is an explanation or hypothesis of how and why a programme (i.e., intervention) is expected to work, given contextual influences and underlying mechanisms of action.^2, 3^ It draws on middle-range theories to provide a level of abstraction that helps in analysing complex data.^2^ |

**References**

1.	National Institute for Health Research [NIHR]. Briefing notes for researchers - public involvement in NHS, health and social care research. Version 2.0: NIHR; 2024 [Available from: <https://www.nihr.ac.uk/documents/briefing-notes-for-researchers-public-involvement-in-nhs-health-and-social-care-research/27371>.

2.	Jagosh J. Realist synthesis for public health: building an ontologically deep understanding of how programs work, for whom, and in which contexts. Annu Rev Public Health. 2019;40(1):361-72.

3.	Pawson R, Greenhalgh T, Harvey G, Walshe K. Realist review – a new method of systematic review designed for complex policy interventions. J Health Serv Res Policy. 2005;10(1_suppl):21-34.

4.	Wong G. Realist Reviews in Health Policy and Systems Research. In: Langlois ÉV, Daniels K, Akl EA, editors. Evidence Synthesis for Health Policy and Systems: A Methods Guide. Geneva: World Health Organization; 2018. p. 52-63.
